# Generating High-Quality Panorama by View Synthesis Based on Optical Flow Estimation

**DOI:** 10.3390/s22020470

**Published:** 2022-01-08

**Authors:** Wenxin Zhang, Yumei Wang, Yu Liu

**Affiliations:** 1 School of Artificial Intelligence, Beijing University of Posts and Telecommunications, Beijing 100876, China; zhangwx@bupt.edu.cn (W.Z.); liuy@bupt.edu.cn (Y.L.); 2 Research Center of Networks and Communications, Peng Cheng Laboratory, Shenzhen 518066, China

**Keywords:** panorama stitching, view synthesis, optical flow

## Abstract

Generating high-quality panorama is a key element in promoting the development of VR content. The panoramas generated by the traditional image stitching algorithm have some limitations, such as artifacts and irregular shapes. We consider solving this problem from the perspective of view synthesis. We propose a view synthesis approach based on optical flow to generate a high-quality omnidirectional panorama. In the first stage, we present a novel optical flow estimation algorithm to establish a dense correspondence between the overlapping areas of the left and right views. The result obtained can be approximated as the parallax of the scene. In the second stage, the reconstructed version of the left and the right views is generated by warping the pixels under the guidance of optical flow, and the alpha blending algorithm is used to synthesize the final novel view. Experimental results demonstrate that the subjective experience obtained by our approach is better than the comparison algorithm without cracks or artifacts. Besides the commonly used image quality assessment PSNR and SSIM, we also calculate MP-PSNR, which can provide accurate high-quality predictions for synthesized views. Our approach can achieve an improvement of about 1 dB in MP-PSNR and PSNR and 25% in SSIM, respectively.

## 1. Introduction

Panoramas have been around for more than one hundred years. Their ability to render a scene in all directions has made them popular in the field of scene visualization and photography. With the rise of the VR (Virtual Reality) industry and the popularity of VR headsets, one of the key enabling elements in creating immersive VR content is panorama, which can provide a compact representation of the scene and more abundant information. Creating a panorama involves a special image stitching task. Image stitching aims at stitching the overlapping region of multiple views collected by multiple cameras with limited angles of view into a wide-angle seamless image, and the panorama requires a viewing coverage of 360°.

Classical image stitching involves the following steps. Starting with image preprocessing, including geometric alignment and photometric correction, the projected transformation model (a homography matrix, generally) between a pair of images is established after feature extraction and feature matching. Then, the projected transformation model is utilized to align one image to another. Finally, the blending algorithm is used to provide the final results. Brown et al.’s AutoStitch [1] was the core algorithm used in commercial software. This used a global homography matric to project the image onto a cylindrical or spherical surface, then synthesized the images using a multi-resolution fusion algorithm. The global homography projection assumed that the overlapping area of the images lay in the same depth plane. Otherwise, the global homography matrix could not align points on different depth planes, which is also the cause of artifacts. Therefore, the APAP [2] of Zaragoza et al. divided the image into dense grids. Each grid was mapped to the canvas of the final stitched image by using the local projection transformation., but this was only suitable for scenes with small parallax. Similar works, such as SPHP [3] by Chang et al., proposed a shape-preserving method from the perspective of shape correction and combined homography warp and spatial similarity to achieve perfect alignment in the overlapping area while maintaining the original viewing angle in non-overlapping areas. Lin et al.’s AANAP [4] also aimed at the shape correction problem of grid distortion, but discarded the constraint term and used global similarity transformation to correct the shape, improving the naturalness and viewing experience of the stitched image. Some methods attempted post-processing in order to eliminate structural distortions such as ghosting or truncation (structural discontinuity) in the overlap area caused by inaccurate registration parameters. The seam-guided [5] of Lin et al. tried to find an optimal seam to partially align the area near the seam to mitigate the distortion caused by unnatural fusion. However, seam-cutting has a huge impact on moving objects. When moving objects pass through the seam, their structures will be broken easily or ghosts will appear around them.

Completely different from traditional stitching methods, since VR capture devices are well-designed (with a regularly arranged camera array), regarding the stitching problem as a viewpoint synthesis problem is more suitable for the generation of high-quality panorama. In previous works on view synthesis, Thatte et al. [6] established a probability model of possible missing points to minimize the disocclusion holes in synthesizing novel views in order to solve the missing areas in the output image of the depth-based view synthesis method. In ref. [7], Zhang et al. addressed view synthesis from a single image. They reorganized the pixels of input view and learned the stereoscopic structure in the multi-scale feature map to synthesize the target view through the learning framework of structure awareness without information about the scene structure, such as depth. The authors of [8] proposed a view-dependent flow-based blending method to generate panoramas with motion parallax in real time.

Motivated by existing works, we propose an omnidirectional view synthesis approach based on optical flow to generate panoramas. In addition to necessary geometric correction and exposure adjustment, our method only relies on the pair of inputs to obtain a synthetic view without scene information or camera parameters. As for feature matching methods, camera intrinsics and extrinsics need to be accurate as they will affect the quality of the stitching. Theoretically, the flow-based method of stitching actually forms a unique projection model for each pixel located in the overlapping area. Therefore, even if the ratio of the overlapping area is insufficient, it will neither affect the novel view results nor destroy the structure of the non-overlapping area.

In this paper, we systematically review existing methods of image stitching and analyze their limitations with respect to panorama generation. According to this goal, we propose a method of panorama generation that is based on optical flow estimation and view synthesis. In detail: (1) we propose a novel optical flow estimation algorithm to obtain translation between views. The obtained flow is essential for subsequence reconstruction and blending. (2) We use the optical flow field to reconstruct the left and right views and combine distance weights and flows with the alpha blending algorithm to synthesize the novel view, which ensures high-quality panorama construction. (3) In the experiments, we show that the proposed approach outperforms previous methods in subjective experience and image quality assessment with the stitched panoramas generated by our method.

## 2. Related Work

### 2.1. Panorama Stitching

Panorama stitching is a technology that stitches images taken from different perspectives together to form a panorama. As for the stitching of an image pair, some homography-based methods [9,10] have been proposed. In these methods, a homography matrix is mainly designed to solve the problems of perspective distortion and shape distortion. In addition, the content-preserving warping is introduced to improve the poor correspondences of low-texture regions in some studies [3,11]. In these studies, the images are first divided into a uniform dense grid mesh. Then, mesh-based warping and optimization are conducted by adding global similarity prior or local similarity transformation to obtain a more accurate alignment. Finally, the overlapping regions of the warped images are blended to obtain a smooth seamless stitched image.

Compared with traditional image pair stitching technologies, panorama stitching improves stereoscopic perception. Therefore, the challenging problem we need to address is that the stitched method can achieve artifact-free within reasonable parallax range to ensure the final panorama is more realistic. In ref. [12], a solution for generating stereo panoramas at a mega pixel resolution was presented and a flow-based up-sampling method was used to resolve the issue of stitching artifacts. Peleg et al. [13] proposed two optical omni-stereo panorama systems to capture images from different perspectives. The left and right panoramas could be spliced by obtaining multiple strips from the camera. While these works also focus on the perception of stitched panorama, flow estimation is at the root of solving parallax and artifacts.

### 2.2. Image-Based Rendering

Image-based rendering aims to synthesize a new viewpoint image of a scene from an input image sequence. Different methods can be used to obtain the information of the target view from the input in different ways. MPEG (Moving Picture Experts Group) divides view synthesis into two categories and gives the official solution VSRS [14]. One of these categories is image-based rendering (IBR), which refers to images from multiple viewpoints. Using 3D-Warping projection, view fusion, interpolation, and other technologies, images from virtual viewpoints can be directly generated [15,16,17,18]. Another method is model-based rendering (MBR), which needs to build an accurate 3D model of a real scene. If a model is built successfully, images can be obtained from any viewpoint [19,20,21,22].

The view synthesis method based on images [15] does not need scene geometry information, but the acquisition equipment of its inputs requires a regular and dense camera grid and the generation of the target view is usually a linear blend of inputs. Chaurasia et al. [16] estimated the depth of each view, mapped the color information and blending weight to the target view according to the depth, and compensated for the inaccuracy of the depth map by the super-pixel method. Zhou et al. [17] introduced a multi-plane image representation that was estimated by a convolutional network for stereo magnification. The image was represented over multiple RGB-α planes, where each plane was related to a certain depth. Novel views can be rendered using back-to-front composition based on this given representation. Flynn et al. [18] used a plane-sweep volume within a network architecture for image-based rendering. A color branch predicted the color values for each depth plane in the target view and another branch predicted the probability.

Some view synthesis methods need to rely on the proxy geometry structure of the scene. Kopf et al. [19] generated 3D proxy geometry by structure-from-motion (SfM) and multi-view stereo (MVS) and achieved a view of the target viewpoint by optimizing Markov Random Field. Aliev et al. [20] described Neural Point-Based Graphics, in which each 3D point was associated with a learned feature vector. These features were segmented into a target view and transformed through a rendering network to synthesize a novel view. However, the feature extractor needed to be re-trained when it was applied to a new scene. Thies et al. [21] used mesh instead of 3D points to embed feature vectors. Sitzmann et al. [22] avoided explicit proxy geometry and projected source images into a neural voxel grid, where each voxel was associated with a trainable feature vector.

In this paper, an optical flow-based view synthesis method is proposed for panorama stitching. In the proposed method, an optical flow estimation algorithm is designed by considering the consistency between pixels of the same world point, and the flow-adjusted version of the left and right views is reconstructed to reduce vertical disparity. Furthermore, we combine optical flow with an alpha-based blending algorithm to synthesize the target view, which ensures a smooth seamless stitched image. Experimental results show that the proposed method performs better in terms of the visual experience.

## 3. The Proposed Method

### 3.1. System Overview

**Figure 1:**
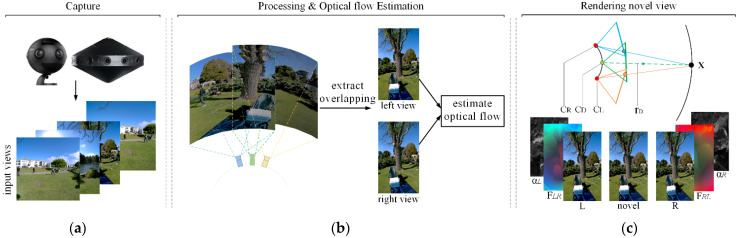
Overview of our approach. (**a**) Capture: experimental data are acquired by these two devices. (**b**) Processing and optical flow estimation: we divide the approximate projection area according to the angular space and calculate the optical flow of each pair of adjacent cameras to establish a dense correspondence (in Section 3.2). (**c**) Rendering: a blending algorithm combining an optical flow and alpha map is proposed to synthesize the view of novel viewpoints (in Section 3.3).

Our method starts with captured data. We use images collected by two products on the market—i.e., Insta360 pro and Facebook surround 360 [23]—to generate our experimental datasets. First of all, because the captured images are affected by the lens of cameras, distortion correction is a necessary pre-processing step in stitching. Then, we need to roughly estimate the overlapping area of the left and right images based on the camera structure. Secondly, we propose a novel optical flow algorithm to calculate the dense optical flow in the overlapping area to achieve pixel-level image matching and use this to reconstruct the left and right views used in the subsequent blending phase. Finally, we combine the flow-based weight with the alpha blending algorithm to synthesize the novel view. We illustrate the steps of our approach in Figure 1, some details will be given in Section 3.2 and Section 3.3.

### 3.2. Optical Flow Estimation

Image-based rendering (IBR) aims to enable the synthesis of novel views of a scene directly from a set of input images. In this process, there are two factors that determine the quality of the synthetic view. First, a center pixel and a small block around it will be mapped to a new position along with the displacement vector of the center pixel, and the mapping error of pixels will cause cracks, holes, or noise. Secondly, due to the occlusion, the background that was originally occluded by the foreground becomes visible in the target view. The question of how to accurately calculate the displacement vector of the same scene point and map it to the corresponding position of the novel view and how to sample the left and right views to make the synthesized view perceptually indistinguishable from reality are two issues that need to be solved. In this paper, we utilize ab optical flow to synthesize the novel view.

**Figure 2:**
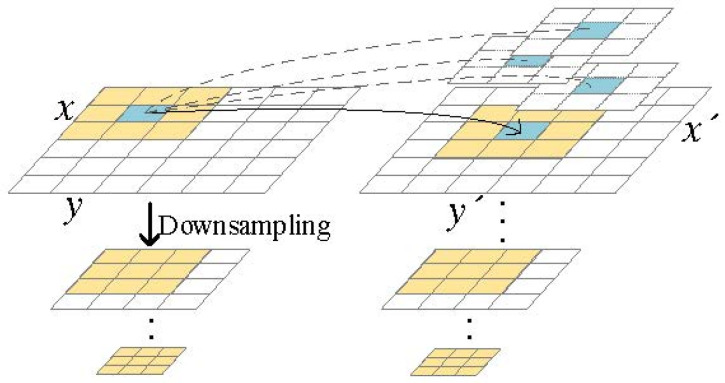
The patch search process in optical flow estimation. The best match is determined by minimizing the $P_{error}$ of a small block around the pixel, and the pixel (x,y) in the current view is mapped to the $\left(x^{\prime },y^{\prime }\right)$ pixel position of the adjacent view.

Optical flow can detect the pixel motion of moving objects and can be widely used in the field of moving object detection and tracking. There are objects with different depth values in the scene described by a set of input images, which makes the relative position of objects in the same spatial position different in various views. We can use optical flow to describe this change. From the two input images $I_{L}$ and $I_{R}$, we synthesize the image $I_{D}$ of the desired view relying on optical flow. We use the optical flow field to approximate the displacement between two views, and the flow field can be well approximated as the inverse depth (parallax) of the scene. Optical flow estimation can be regarded as point-by-point per-pixel matching. It returns a displacement vector for each pixel of the original image and maps it to a new position in the reference images. The pixel matching process is depicted in Figure 2.

First, we separate the gray components and alpha components of the input images (.png format images contain alpha channels; other formats need to be converted into .png). The alpha maps of two images are used to adjust the intensity of the image (gray components) to further alleviate the spatial gray difference between the two images after exposure adjustment. Additionally, this process is explained by Equation (1). Next, we downsample the images by scaling the image height and width by a factor of 2 as the inputs of the image pyramid structure to make this process faster and more stable. By gradually downscaling the image, the basic assumption of optical flow with small displacement is satisfied so as to deal with the large motion of objects in the scene. Finally, we perform patch searching for both gray and alpha pyramids at the same time from the top level of the pyramids with the lowest resolution. Here, we set the size of the patch to 2 N +1, setting Ν to 2. We calculate the intensity error of the pixel value in the patch recorded as $P_{error}$, as depicted in Equations (2)–(4):

(1) $$\;I_{R}=I_{R}\left(\;x,y\;\right)\times \frac{\sum I_{L}\left(x,y\right)\times \alpha_{L}\left(x,y\right)\times \alpha_{R}\left(x,y\right)}{\sum I_{R}\left(x,y\right)\times \alpha_{L}\left(x,y\right)\times \alpha_{R}\left(x,y\right)}$$

(2) $$I_{abs}=\overset{N}{\underset{v=-N}{\sum }}\overset{N}{\underset{u=-N}{\sum }}\left|I_{L}\left(x_{L}+u,y_{L}+v\right)-I_{R}\left(x_{R}+u,y_{R}+v\right)\right|$$

(3) $$\alpha =\overset{N}{\underset{v=-N}{\sum }}\overset{N}{\underset{u=-N}{\sum }}\alpha_{L}\left(x_{L}+u,y_{L}+v\right)\times \alpha_{R}\left(x_{R}+u,y_{R}+v\right)$$

(4) $$P_{error}=\frac{I_{abs}}{\alpha }\cdot \left[1+2\times L_{2}\left(x_{R}-x_{L},y_{R}-y_{L}\right)\right]$$

$I_{abs}$ is calculated as the sum of absolute differences (SAD) of intensity in the search box; α is the dot product of alpha values in the patch; $P_{error}$ is calculated as the second norm of displacement vector in the *x* direction and *y* direction plus the ratio of SAD to alpha, which matches the pixel characteristics in the patch more accurately.

Taking the occlusion caused by parallax and the different orientation of the image into account, only searching within the 5 × 5 tile does not enable us to obtain the correct correspondence for nearby objects. We expand the search box as a rectangle to update the initial match. The rectangle extends ortho to each side of the search direction (left or right), and the scope around $I_{L}\left(x,y\right)$ is limited to:

(5) $$Rect\left(-k,-\left(\frac{k}{kRatio}+\frac{1}{2}\right),k+1,2\left(\frac{k}{kRatio}+1\right)\right)$$

**Figure 3:**
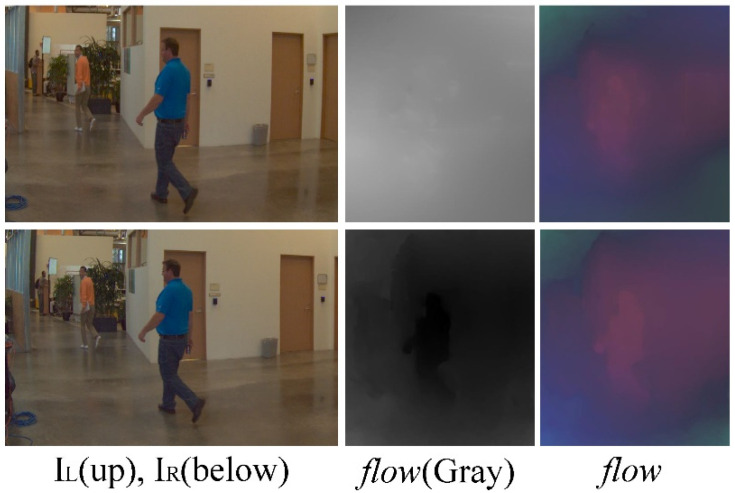
Rectangular search can better detect the foreground and keep its outline. The upper are the optical flows obtained by a 5 × 5 patch, and the lower are the results of the extended rectangular search box. A color wheel is used to color the gray version of the flow to give an obvious effect.

In Equation (5), the four items of Rect are the coordinates of the upper left point, width, and height of the searchbox, respectively. Here, k is a constant ranging from 0 to 1 that determines the degree of expansion and kRatio determines the aspect ratio of the search box. Rectangular search considers the depth varies with scene content, which is of benefit to the foreground content (as seen in Figure 3). We use the best match with the smallest error in the expanded box, which signifies the best correspondence. The displacement vector of the point (x,y) is described as (u,v), which is treated as flow.

After patch searching, we obtain the primary matching of pixels depending on the similarity of the gray neighborhood. However, the gray variation is not obvious for the smooth areas (there may be some silhouettes and sloped surfaces) in the image. In order to maintain this edge information, more advanced features besides gray information are added in the process of calculating the matching error to improve the primary flow field. Gradient norm errors in the *x* and *y* directions of images are considered. We sweep the flow from two directions to obtain the final optical flow field F. The description of the details is given in Algorithm 1. In this step, we introduce the adjacent points at the four positions of up, down, left, and right to finally determine whether the current displacement vector indicates the optimal pixel correspondence. When obtaining the final optical flow field F, the transparency information of images is used to weight the flow and its gaussian blur version so as to improve the robustness of the optical flow estimation.
**Algorithm 1.** Calculate the optical flow field.**Input:**$I_{L}$,$\;I_{R}$—the grayscale images of the left and the right views $\alpha_{L}$,$\;\alpha_{R}$—the alpha maps of the left and the right views $dx_{L}$,$\;dy_{L}$,$\;dx_{R}$,$\;dy_{R}$—gradients in two directions flow, blowflow—the flow and its gaussian blur version **Output:** F—the final optical flow field 1.  **function** errorfunction(x,y,flow.at(x,y))2.      $\left(x^{\prime },y^{\prime }\right)\leftarrow \left(x,y\right)+flow.at\left(x,y\right)$3.      $\left(i_{L}x,i_{L}y\right)\leftarrow \left(dx_{L}.at\left(x,y\right),dy_{L}.at\left(x,y\right)\right)$4.      $\left(i_{R}x,i_{R}y\right)\leftarrow \left(dx_{R}.at\left(x^{\prime },y^{\prime }\right),dy_{R}.at\left(x^{\prime },y^{\prime }\right)\right)$5.      diff←blurflow.at(x,y)−flow.at(x,y)6.      $error={\left\|(i_{L}x-i_{R}x,i_{L}y-i_{R}y)\right\|}_{2}+\sqrt{diff⊙diff}$7.  **end function**8.  **for** x from 0 to $I_{L}$.width **do**9.      **for** y from 0 to $I_{L}$.height **do**10.            currentError←errorfunction(x,y,flow.at(x,y))11.            (0,1),(0,−1),(−1,0),(1,0) successively assign to (i,j)12.            error←min[errorfunction(x,y,flow.at(x+i,y+j))]13.            **while** error<currentError **do**14.                  flow.at(x,y)←flow.at(x+i,y+j)15.            **end while**16.      **end for**17.  **end for**18.  $k=1-\alpha_{L}.at\left(x,y\right)\times \alpha_{R}.at\left(x,y\right)$19.  F(x,y)←(1−k)×flow.at(x,y)+k×blurflow.at(x,y)

**Figure 4:**
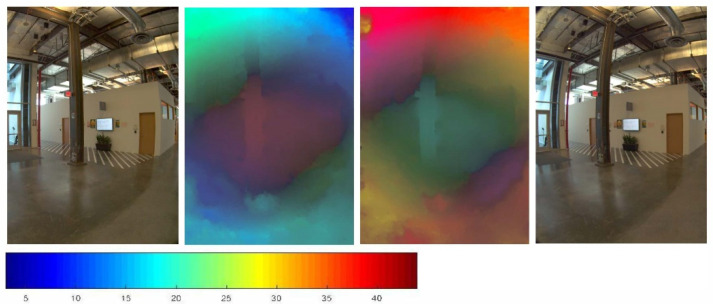
The optical flow approximates the inverse depth of the scene. A color bar of the optical flow field is shown below, and the value represents disparity in the x direction.

Algorithm 1 describes the calculation of the optical flow from the left view to the right view. Taking $I_{L}$ as a reference, we need to find the correspondence in $I_{R}$ and convert it accordingly to obtain the optical flow in the other direction. Figure 4 shows the result of our optical flow calculation. From the left to right are the left view, $F_{LR}$, $F_{RL}$, and the right view. We use colorwheel to color the displacement vector because the optical flow field describes the displacement vectors from two opposite directions and shows the difference in the color temperature.

### 3.3. Reconstructed View-Based Blending Algorithm

**Figure 5:**
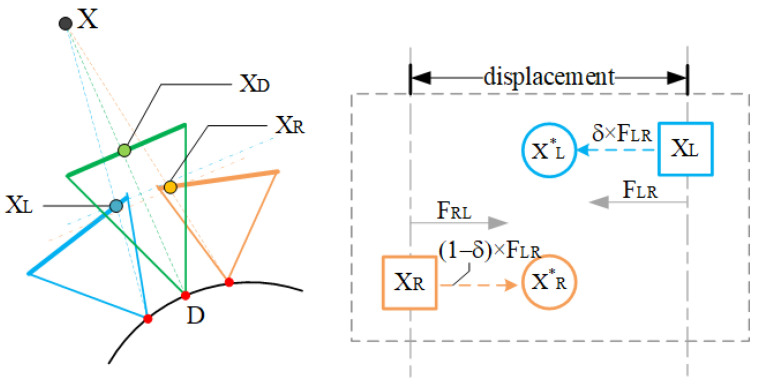
Get the reconstructed views by warping pixels depending on optical flow.

Due to parallax and camera orientation, the overlapping areas of the left and the right images are not perfectly matched and blending directly based on distance weights will cause artifacts. By calculating the optical flow, we obtain the displacement between the overlapping area on the 2-dimensional plane. In order to synthesize the image of the target viewpoint, we consider obtaining an intermediate pattern depending on the optical flow to improve the corresponding accuracy of the overlapping area. Based on the intermediate pattern, we can synthesize the image of the target view $I_{D}$ by using the spatial position relationship and image transparency; the geometric model is described in Figure 5.

The world point X projects to $x_{L}$ and $x_{R}$ in the left and the right views, respectively. We interpolate a novel viewpoint D and synthesize its imaged view $I_{D}$, where $x_{D}$ is the scene point X projected in $I_{D}$. The dotted blue and yellow lines on the left in Figure 5 depict the plane-induced depth mismatch. We use optical flow fields $F_{LR}$ and $F_{RL}$ to reconstruct the left and the right views to compensate for the truncation and artifacts caused by this mismatch. We adjust the optical flow amplitude based on the position information to determine the coordinates of sampling points $x_{L}^{*}$ and $x_{R}^{*},$ which are needed for image reconstruction. The amplitude adjustment parameter δ is as follows:

(6) $$\delta =\mathrm{sigmod}\left(\frac{scale}{W}\times \left(pos-\frac{W}{2}\right)\right)$$

where scale is an adjustable parameter that controls the degree of change in the linear region of the sigmoid function curve. The larger the scale is, the more the displacement of the optical flow field plays a role in the sampling coordinates. W refers to the pixel width of the image, and pos refers to the column position of the pixel. For the left view, the left part is only slightly tuned by the $F_{LR}$ according to the value range of the sigmoid function and is almost the same as the left side of the original image. Additionally, the right part is tuned a lot dependent on $F_{LR}$. The coordinates of the left and the right views warped by optical flow are:

(7) $$x_{L}^{*}=x_{L}+\delta \times F_{LR}\left(x_{L}\right)$$

(8) $$x_{R}^{*}=x_{R}+\left(1-\delta \right)\times F_{RL}\left(x_{R}\right)$$

**Figure 6:**
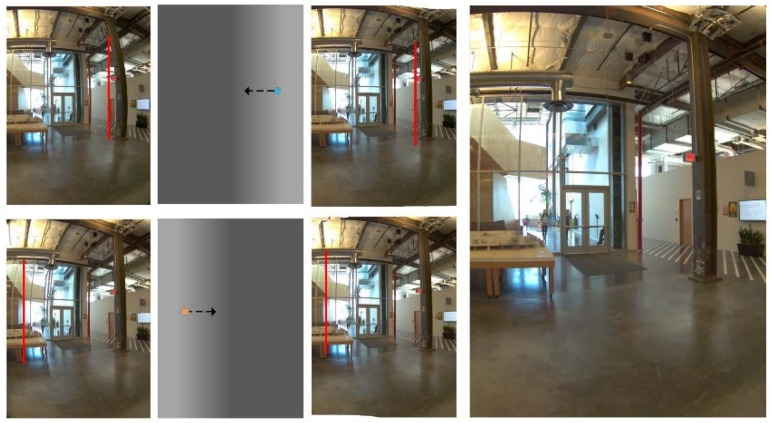
Optical flow adjustment and the synthesis of the novel viewpoint.

Figure 6 shows the left and the right views after optical flow adjustment. The top and the bottom of the first column are the original left and right views, and the second column is the value map of the adjustment function according to amplitude adjustment parameter. The brighter part indicates that the original view is adjusted a lot by the optical flow. The third column is the reconstructed left and right viewpoint images, depicting that the vertical distortion with red line is corrected after the optical flow adjustment. The last column is the synthesized view.

Vertical distortion is most noticeable for nearby scene objects and affects the viewing experience of users. Additionally, the synthesized image combines views from multiple perspectives, which leads to distorted scene objects in novel viewpoints. We use the alpha value, which indicates the transparency of the pixel, to blend the reconstructed views which can tolerate a certain degree of parallax. If the transparency is effectively controlled, the artifacts can be suppressed to a certain extent.

The alpha values of the left and the right images are represented by $\alpha_{L}$ and $\alpha_{R}$, respectively. Here, we take $L_{1}=\delta$ and $R_{1}=1-\delta$ as the optical flow weights. In fact, $L_{1}$, $R_{1},$ and the alpha value of the pixel are employed to obtain the base $K_{L}$ and $K_{R}$ of the transparency weight value represented by $L_{2}$ and $R_{2}$, as shown in Equations (9) and (10):

(9) $$K_{L}=e^{\left(k\times L_{1}\times \alpha_{L}\right)}$$

(10) $$K_{R}=e^{\left(k\times R_{1}\times \alpha_{R}\right)}$$

where k is an adjustable coefficient. According to the alpha blending algorithm, the sum of the transparency weights of the two images is 1. Therefore, $L_{2}$ and $R_{2}$ are obtained according to Equations (11) and (12):

(11) $$L_{2}=\frac{K_{L}}{K_{L}+K_{R}+\varepsilon }$$

(12) $$R_{2}=\frac{K_{R}}{K_{L}+K_{R}+\varepsilon }$$

where *ε* is an extremely small positive number.

Finally, we use the difference in the absolute value of the pixel at the same position, which is referred as d, to evaluate the importance of these two weights. Additionally, we use ratio to express this importance:

(13) ratio=tanh(d)

where tanh ensures that when the difference of pixel values is large, the transparency weights of the image play a leading role in the process of blending, and we are able to obtain a compromise between the optical flow weight and alpha weight. Then, we obtain the final blending weights of the reconstructed left and right images according to Equations (14) and (15)

(14) $$w_{L}=L_{1}\left(1-ratio\right)+L_{2}\times ratio$$

(15) $$w_{R}=R_{1}\left(1-ratio\right)+R_{2}\times ratio$$

To synthesize the novel view $I_{D}$, we combine the pixels sampled at $x_{L}^{*}$ and $x_{R}^{*}$ from $I_{L}$ and $I_{R}$, respectively. Additionally, we perform a convex combination:

(16) $$I_{D}\left(x_{D}\right)=w_{L}\times I_{L}\left(x_{L}^{*}\right)+w_{R}\times I_{R}\left(x_{R}^{*}\right)$$

Note that $w_{L}+w_{R}=1$ according to the fusion rule of alpha blending.

## 4. Experimental Results

### 4.1. Datasets

Since there is no benchmark dataset in the field of panorama stitching, we use real image datasets ‘scene1’ and ‘scene2’ captured by the Facebook surround360 device and ‘fisheye’ from the Insta360 device to build a panorama and use the public light field datasets HCI and ‘Teddy’ from Middlebury [24,25] to conduct the comparison between methods. In the ablation experiments, the stereo/flow 2012 dataset of KITTI [26] is used to evaluate each component of the algorithm. The camera array of Surround360 is composed of 14 cameras on the fixed camera rig with the same intrinsics at equal intervals. The optical centers of the cameras are in the same horizontal plane. The resolution of the images is 2048 × 2048. The Insta360 camera rig is arranged by 6 fisheye cameras. We expand the fisheye images and extract the central area as the inputs according to the Equi-Rectangular Projection (ERP) projection theory, where the size of images is 1360 × 2042. Each scene of the HCI light field dataset is composed of 81 images by a 9 × 9 camera array with a resolution of 768 × 768. Each scene in the Middlebury dataset is composed of 6 views, and the resolution is larger than 600 × 500. The KITTI dataset contains stereo pairs and is from the real scene of autonomous driving, which is commonly used to evaluate vision algorithms.

Scene1 is a building interior scene that does not contain many complex textures but rather quantities of vertical or horizontal object edges. A good method should keep the original structure in the vertical direction. Scene 2 is a spacious outdoor scene with complex texture objects such as grass and branches. This kind of texture is prone to unsatisfactory stitching distortion artifacts, which poses a great challenge to the effectiveness of the methods. Moreover, Insta360 is an indoor scene with varying depth, and the images are in the ERP projection format, which means that the scene is deformed. Light field data [24] are acquired by a structured camera array and can be seen as a narrow baseline image pair. On the contrary, ‘TEDDY’ is an image pair with a wide baseline. KITTI data are collected by self-driving vehicles, usually including buildings, vehicles, roads, and trees. These real scenes have covered most of the complex textures to evaluate the performance of our method comprehensively.

### 4.2. Ablation Study

**Figure 7:**
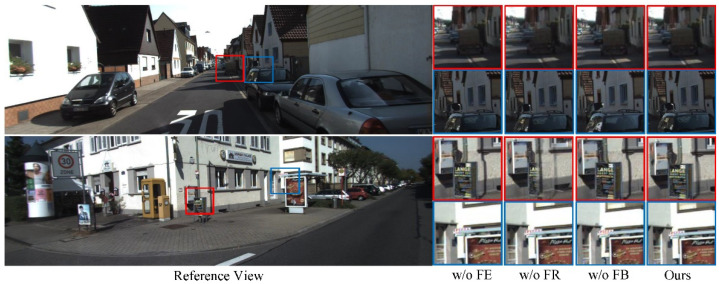
Taking the right view as the reference view for the ablation experiments to verify the effectiveness of flow estimation (FE), flow-based reconstruction (FR), and flow-based blending (FB).

Flow Estimation (FE): The first step of our approach is optical flow estimation, which represents the correspondence between the left and right views. To verify the importance of this module, we compare the performance in the case of ‘w/o FE’ with ‘Ours’ in Table 1 and Figure 7. Concretely, we implement the optical flow part of surround360 [23] to calculate optical flow and replace our proposed optical flow estimation in the ‘w/o FE’. From the comparison of the subjective results, it can be seen that the two are close. However, our method keeps the shape at the vertical and horizontal edges of the second scene. Therefore, ‘Ours’ can achieve a higher peak signal to noise ratio (PSNR) and structural similarity (SSIM), which means that our results have a lower noise error and higher structural similarity, as shown in Table 1.

Flow-based reconstruction (FR): Due to parallax, the baseline and rough estimation of the overlapping area, blending directly based on distance will lead to bad results. Note that we have calculated the displacement between pixels through optical flow estimation.

We first adjust the optical filed depending on the positional information and then fine-tune the two views using the adjusted flow field to obtain an intermediate pattern between the left and right views that can reduce artifacts in subsequent blending. To verify the necessity of this process, we eliminate the process of flow adjustment in the case of ‘w/o FR’. As illustrated in Figure 7, ‘w/o FR’ shows a discontinuity of structure in the part circled in red.

Flow-based blending (FB): In this work, we utilize the alpha value to design our blending algorithm. The alpha map represents the visibility of each pixel of an image and is widely used in view synthesis. In this paper, a dual-weight blending algorithm based on optical flow is designed to determine the weights of the left and the right views in the final sampling. Comparing our method with the traditional alpha blending algorithm ‘w/o FB’, we find that the results of ‘w/o FB’ and ‘Ours’ are almost similar in some local parts. While there is a certain degree of deformation in the parts circled in blue in the first scene and red of the second scene in ‘w/o FB’, this can be controlled by our method, as shown in Figure 7.

### 4.3. Viewing and Analysis

**Figure 8:**
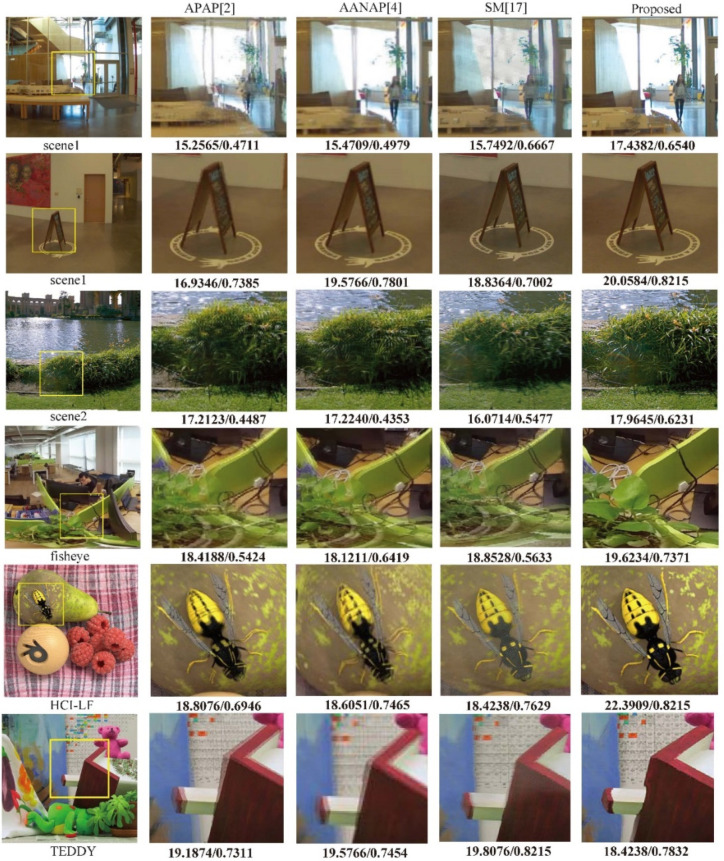
The comparison of the results obtained from other methods proposed for use in several scenes. The values below the images are PSNR and SSIM, respectively.

**Figure 9:**
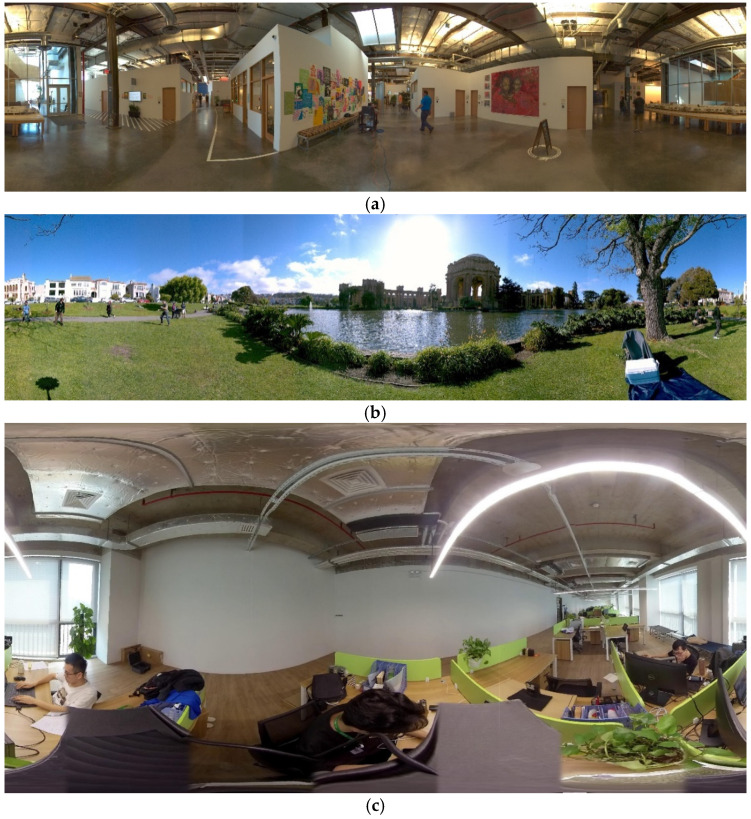
‘Ours’ panoramic images. (**a**) scene1; (**b**) scene2; (**c**) fisheye.

For datasets that cannot form a panorama, subjective synthetic view results are displayed to ensure the rationality of the experiment in Figure 8. For the datasets that can form the panorama, we show the formed panoramas and calculate the image quality assessment metrics over the image sequence in addition, as shown in Table 2 and Figure 9. We compare our algorithm with other methods, such as APAP [2], AANAP [4], and the view synthesis method SM [17], from the left to the right in Figure 8. The first two rows are the contents of scene1, the next row is scene2, and the last scene is fisheye data. The next scene is HCI light-field data from [24], and the last scene is TEDDY from the Middlebury datasets [25]. The values under the figures are PSNR and SSIM, respectively.

In scene1, our method achieves better performance on the vertical edges of the building, while other methods show ghost and stitching distortions. In the fisheye scene, the contrast methods have artifacts and truncation effects on the green baffle and the black cable. On complex textures such as tree branches, our approach can also perform well without artifacts. While SM [17] obtains the best performance on TEDDY, our approach shows an irregular edge due to the large parallax that optical flow adjustment cannot overcome. Figure 1 shows the panoramas generated by our approach on three panoramic datasets.

We also calculate image quality assessment metrics on scene1, scene2, and fisheye data, as shown in Table 2. The difference between the values of a single image pair may be relatively large, so we evaluate the metrics on the whole dataset. The result of the single image pair is shown below the picture because it cannot form a complete panorama. We calculate MP-PSNR [27], PSNR, and SSIM to evaluate these approaches. The SSIM value gain of our method is close to 0.1 in scene1, while the MP-PSNR and PSNR gains are close to 1dB in the fisheye scene. Experimental results show that our results are better than the comparison methods to a certain extent in terms of both subjective visual perception and objective assessment metrics.

## 5. Conclusions

In this paper, we proposed a view synthesis approach based on optical flow to solve the problem of panorama stitching. First, we estimated the optical flow field to match images that had a certain robustness with overlapping and non-overlapping areas. Secondly, the obtained optical flow field was used to warp the left and right views to obtain the reconstructed views for the subsequent blending stage. We showed that the reconstruction version of views could reduce the vertical distortion of the original image. In the rendering process, we considered the optical flow and the alpha value of pixels to interpolate each pixel in the novel view. Compared with existing methods, our flow-based view synthesized method was able to eliminate most artifacts and structural distortions.

## Figures and Tables

**Table 1:** Ablation studies on flow estimation by Surround 360 [23], flow-based reconstruction, and flow-based blending.

Pipeline	MP-PSNR	PSNR	SSIM
w/o FE	26.7732	21.5039	0.6301
w/o FR	24.6985	18.4631	0.5833
w/o FB	25.9854	18.7952	0.5711
**Ours**	**28.0473**	**23.2075**	**0.7027**

**Table 2:** MP-PSNR [27], PSNR, and SSIM comparison of the proposed method and [2,4,17]. The bold items indicate which method gets the better performance in each scene.

Datasets/ Models	APAP [2]			AANAP [4]			SM [17]			Proposed		
	scene1	scene2	fisheye	scene1	scene2	fisheye	scene1	scene2	fisheye	scene1	scene2	fisheye
MP-PSNR [27]	25.0831	24.1017	24.1743	25.9847	24.1833	24.5380	27.1593	25.0037	24.0649	27.1482	25.1794	24.5882
PSNR	17.3444	20.1031	20.1295	18.8771	18.1437	18.1437	**21.2529**	19.5766	20.7835	21.0021	20.1295	**21.2200**
SSIM	0.4015	0.6100	0.6218	0.4275	0.6463	0.7078	0.4979	0.7167	0.5732	**0.5518**	**0.7411**	**0.7914**

## Data Availability

Not applicable.
